# Medical Certificates

**Published:** 1897-02-06

**Authors:** 


### The Hospital

A Journal of

The Medical Sciences and Hospital Administration.

Vol. XXI.—No. 541.] [Feb. 6, 1897.

### Medical Certificates.

The stupid person always assumes that "that which has been is that which shall be," and which ought to be. In times past it might have been said of vast numbers of medical certificates that they were not worth the paper they were written upon, and it came about that the term medical certificate became a byword and an object of ridicule and contempt It is assumed very frequently that this is so still. If a man wishes to avoid sitting on a jury he straightway applies to his doctor for a certificate of illness, and expects the doctor, with all the circumstantiality of careful grammar, and one or more medical terms ending in "itis," to demonstrate that his client is exceedingly ill, and quite incapable of performing the duties of a juryman. It is of no moment that the "patient" intends to go to his club, dine out, and fulfil all his professional and social duties in blissful disregard of his serious illness and all the direful consequences to which it may give rise. Notwithstanding all this, he does not hesitate to ask for a certificate! This is but a solitary instance; but it is well known that, to the public, a medical certificate seems one of the grand panaceas for almost every kind of social or personal inconvenience into which either man or woman may fall.

Some severe remarks were made the other day on this matter of certificates by Judge Emden at the Lambeth County Court. We do not wish to place a brother practitioner on a "bad eminence" by giving his name. But Judge Emden's remarks are of importance, expressing as they do the feeling entertained on the subject by most members of the medical profession. "Never in all my experience," declared the Judge, "have I heard or seen such an extraordinary document from a professional man. It is the duty of medical men when called upon for a report to keep themselves to the medical aspects of the case, and not to assume the functions of an advocate in arguing the merits of the case. If this doctor's example were followed medical men would cease to be of any use to judges and juries; they would, indeed, hinder rather than help the course of justice."

Now we are well aware that judges, and the legal profession generally, appear to take a heartfelt pleasure in "snubbing" the members of the medical profession; but for this very reason should the doctor, knowing what a keen critic the lawyer can be, always keep strictly to fact, and legitimate inferences therefrom, in any certificate he may give. It is seldom or never a policy which commends itself to worldly wisdom for a man to "give himself away." The doctor who displays animus of any kind in such a certificate, gives himself away absolutely; and if his certificate happens to come into the possession of a lawyer, it is ten to one that the doctor is made to repent bitterly of his folly. For our part we think he is served right; and we do not feel that we can blame the lawyer at all.

The question, indeed, is one of great importance; and it is imperative that a proper sense of the responsibility attaching to all kinds of certificates shall be made to prevail. The doctor is a man of science; he knows what he is about when he examines a man and reports upon his condition, and the public knows that he knows, and if a doctor should give a certificate which is obviously foolish or not true, the public look upon him not as a fool but as a fraud. It is here where the mischief lies. The giving of improper certificates is not only wrong in itself, but the public is thereby injured and defrauded because it cannot obtain the true and genuine knowledge needful for its guidance, notwithstanding that it knows such knowledge to be in the possession of the medical profession.

There are many members of the public who think that the doctor will do anything "for a fee." They know the lawyer will; they see him every day, in his very highest representatives, selling his eloquence, his morality, his virtuous indignation, anything he has or can bring together, to the first applicant who can pay his fee; and they cannot see why the doctor should not do the same. It would be the easiest thing in the world to retort upon Judge Emden himself in these very terms. But that would be entirely beside our purpose. However it may be with lawyers, it is absolutely imperative that medical men and men of science should always certify the truth, and nothing but the truth. The doctor, by the nature of the case, cannot be an advocate. At the bedside he asks for the truth from his patient, and he tells the truth to his patient. Nothing else is of any avail whatever. In like manner, in all his public duties, the public has a right to expect what the medical profession in general is always willing to acknowledge—the unchallengeable obligation of the truth and the truth only.

We insist on the one hand upon the necessity for a universal recognition of the high standard of professional honour already accepted among the majority of medical men; and, on the other, upon the obligations which every man in a civilised and cultured community ought to recognise, of doing the utmost that is in him, by simplicity and straightforwardness, to secure for himself and all his fellowmen the incalculable blessings of fairness, and the observance of that rule of the highest civilisation which insists that every man ought to do as he would be done by.

